# Case of cutaneous metastasis from presumed poorly differentiated thyroid carcinoma: a case report

**DOI:** 10.1186/s13256-025-05565-9

**Published:** 2025-10-17

**Authors:** Nafkot Girum, Ahmed Issa, Hiwot Tesfaye, Selamawit Worku, Selamawit Tamene, Tesema Etefa

**Affiliations:** 1 Department of Dermatovenerology, Faculty of Medicine, College of Health Sciences, Addis Ababa University, Addis Ababa, Ethiopia; 2 Department of Dermatology & Venereology, Yekatit-12 Hospital Medical College, Addis Ababa, Ethiopia; 3 Department of Dermatology, St. Paul’s Hospital Millennium Medical College, Addis Ababa, Ethiopia; 4 Department of Biomedical Science, College of Medicine, Institute of Health, Jimma University, Jimma, Ethiopia

**Keywords:** Cutaneous metastasis, Poorly differentiated, Thyroid carcinoma, Case report

## Abstract

**Introduction:**

Cutaneous metastasis from internal malignancy is relatively uncommon and may rarely be the presenting sign of an internal malignancy. The current investigation included a case of cutaneous metastasis from presumed poorly differentiated thyroid carcinoma at Addis Ababa University (Black Lion Hospital) in Addis Ababa, Ethiopia. We present this case report to help with the early diagnosis and treatment of cutaneous metastasis from presumed poorly differentiated thyroid carcinoma.

**A case report:**

A 28-year-old Ethiopian female presented to the Dermatology Department at the Faculty of Medicine at Addis Ababa University (Black Lion Hospital) in Addis Ababa with multiple, round, ulcerated nodules on the scalp for 6 months and small round skin-colored subcutaneous nodules on the neck, abdomen, back, and chest. She also had a mobile, firm, nontender, and multinodular anterior neck mass that moved upon swallowing. The patient was diagnosed with cutaneous metastasis from presumed poorly differentiated carcinoma, considering the top differential diagnosis, thyroid carcinoma. Owing to a lack of immunohistochemistry and inability to perform a thyroid biopsy, we could not explicitly state that this was cutaneous metastasis from thyroid carcinoma. Treatment followed the regimen appropriate for the primary metastatic malignancy. Local treatment options include imiquimod cream, liquid nitrogen cryotherapy, photodynamic therapy, excision, carbon dioxide laser therapy, pulsed dye laser therapy, topical and intralesional chemotherapy, and cytokines. The patient deferred follow-up and passed away after 3 months of the initial presentation.

**Conclusion:**

A multidisciplinary treatment approach, including medical, surgical, radiation, oncologists, and mental health care providers. Cutaneous metastases are typically indicative of disseminated disease and portend a correspondingly poor prognosis.

## Introduction

Cutaneous metastasis from internal malignancy is relatively uncommon and may rarely be the presenting sign of an internal malignancy. It demonstrates the spread of malignant cells to the skin by metastatic spread of the distant primary tumor [1] and occurs in 1–10% of patients with metastatic disease and 0.5–1% of cases [2]. The scalp is the most common site of thyroid carcinoma metastasis to the skin. The estimated mean survival after the diagnosis of cutaneous metastases is 50% at 6 months, and survival is only about 3 months in patients with disseminated skin metastases [3].

The lungs and the bones are the most common sites for distant metastasis [4]. Risk factors for distant metastases include male sex, advanced age, histologic grade, completeness of surgical resection of the primary tumor, extra thyroidal extension, and lymph node metastasis on initial examination [5–7]. The gross appearance of cutaneous metastases varies, with reports describing slow-growing nodules, erythematous papules, and ulcerated scrotal lesions. Lesions may not be painful or irritating, and they can be mistaken for a pimple or other benign lump. The current investigation included a case of cutaneous metastasis from presumed poorly differentiated thyroid carcinoma at Addis Ababa University (Black Lion Hospital) in Addis Ababa, Ethiopia. It improves patient outcomes, increases knowledge of the illness, maximizes the use of resources, and fosters health equity in low-resource populations. We present this case report to help with the early diagnosis and treatment of cutaneous metastasis from presumed poorly differentiated thyroid carcinoma.

## A case report

A 28-year-old Ethiopian female presented to the dermatology department at the Faculty of Medicine, Addis Ababa University (Black Lion Hospital), Addis Ababa, Ethiopia, with multiple, round, ulcerated nodules on the scalp for 6 months and small, round, skin-colored subcutaneous nodules on the neck, abdomen, back, and chest. The lesions were painless but had associated intermittent itching and burning sensations. In addition, she was easily fatigued and experienced recent significant weight loss and night sweats. On presentation, she had an anterior neck mass that was painless and moved upon swallowing. She had no previously diagnosed known chronic medical illness and no history of abdominal pain, nausea, vomiting, or yellowish discoloration of the eyes. In addition, she had no headache, bone pain, or recent behavior change; no history of herbal or traditional medication use; no history of previous medical treatment; and no family history of similar condition.

**Fig. 1:**
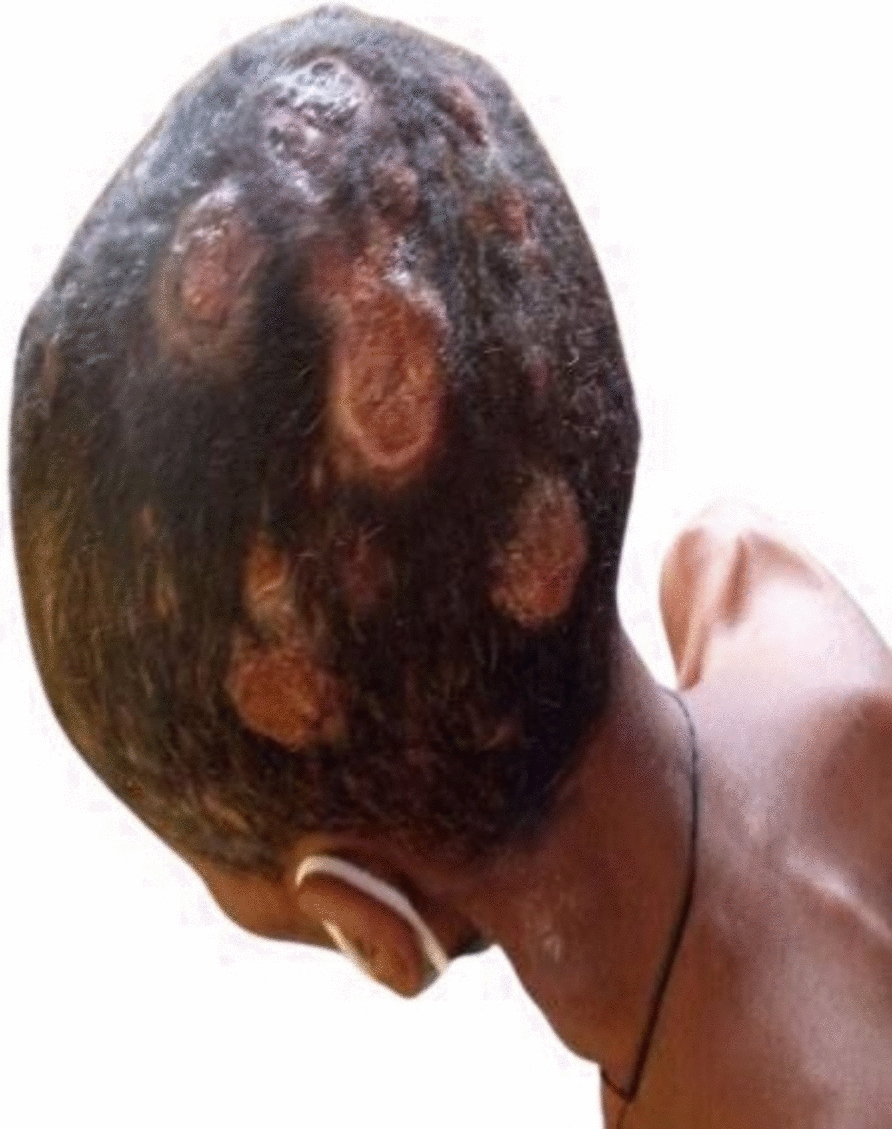
Multiple skin-colored, nontender subcutaneous nodules and ulcerated plaques on the scalp

**Fig. 2:**
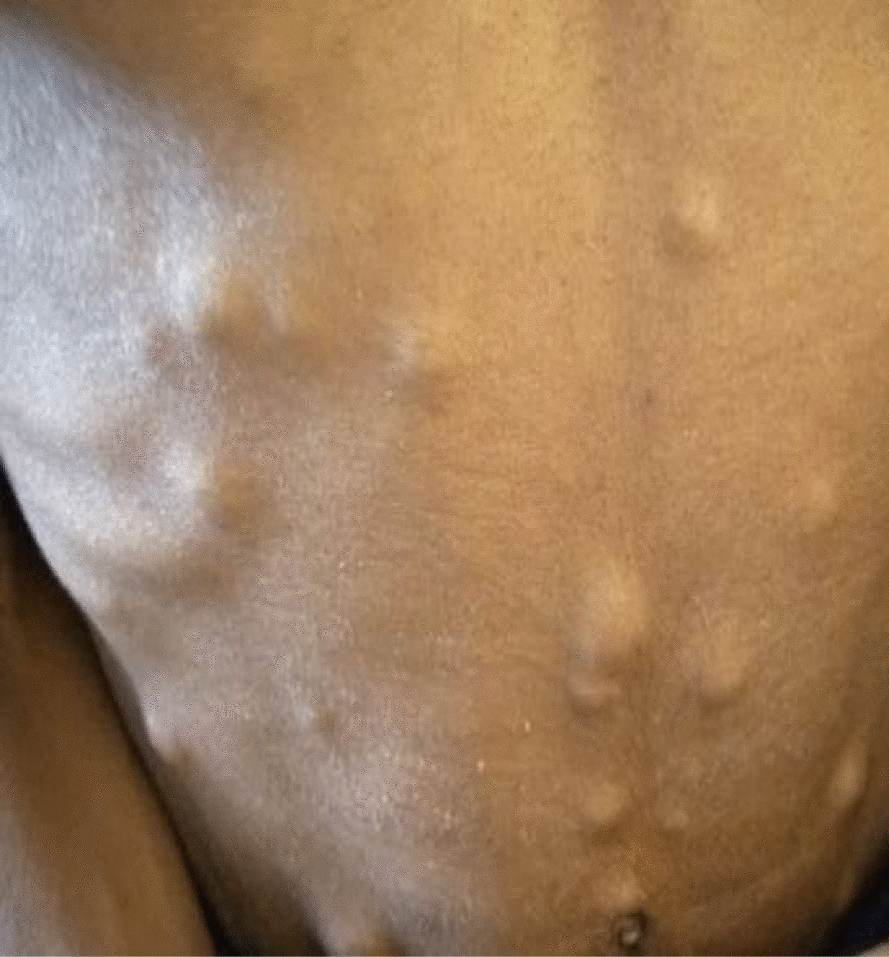
Multiple skin colored, mobile, nontender subcutaneous nodules

**Fig. 3:**
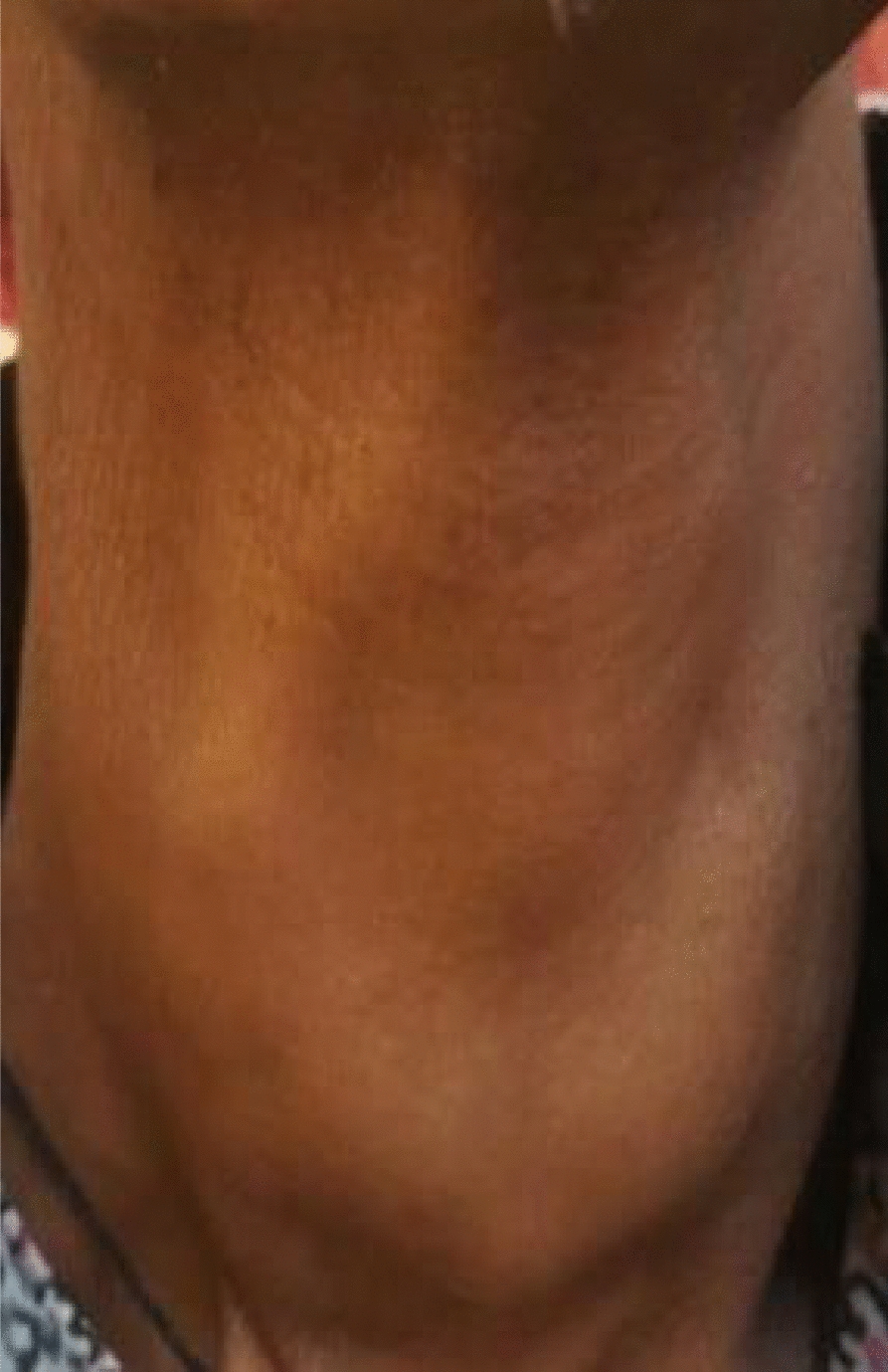
Firm, nontender anterior neck mass

On physical examination, she was cachexic and had multiple ulcerated nodules, plaque, and tumorous lesions with overlying hemorrhagic crust on the scalp, with areas of alopecia overlying the lesions, as well as multiple firm skin-colored subcutaneous nodules on the chest, back, neck, axilla, and bilateral breasts. There was a mobile, firm, nontender multinodular anterior neck mass that moved with swallowing (Figs. 1, 2, 3). She had no lymphadenopathy in all accessible areas. There was no palpable mass on the breasts, abdomen, or pubic area. The size of the lymph nodes has not been mentioned because she had no lymphadenopathy. The laboratory investigations (complete blood count (CBC), liver function tests (LFT), renal function tests (RFT), and thyroid function test(TFT) were all in the normal range. Chest X-ray showed a 4.3 cm × 3.5 cm oval well-defined mass in the left lower lung zone.

## Microscopic features

**Fig. 4:**
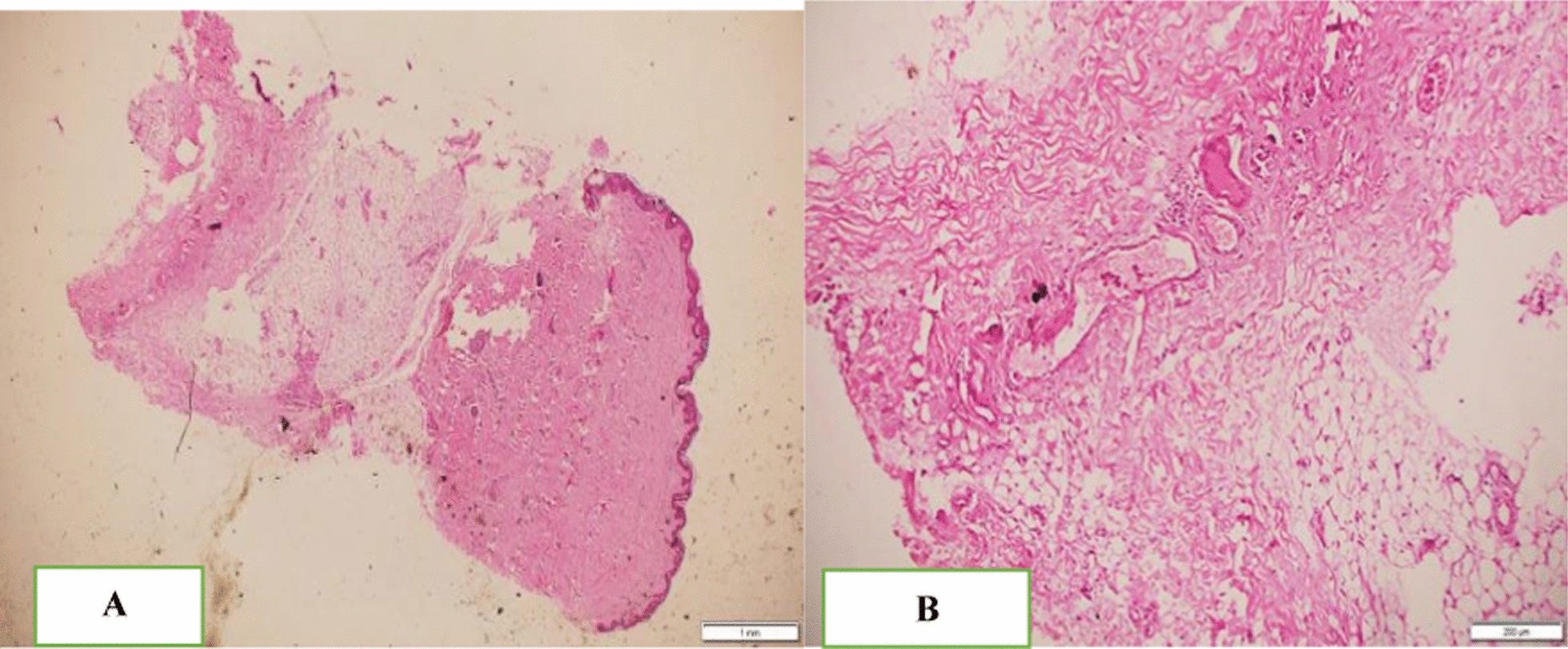
Histopathology feature of subcutaneous nodule. **A** Low-power view at 4× showing bland-looking maturing epidermis and dermis. Focal deposits of small, round blue cells in subcutaneous tissue. **B** High-power view at 40× showing focal deposits of hyperchromatic round-to-oval cells with eosinophilic round cytoplasm arranged in cohesive aggregates within the subcutaneous tissue and focal areas of vascular invasion

Fine-needle aspiration cytology (FNAC) from thyroid: Smears from scalp, breast, thyroid, and soft tissue showed cellular aspirate composed of cohesive pleomorphic round-to-oval cells with abundant bluish cytoplasm at areas forming hemorrhagic background. With a conclusion of presumed poorly differentiated carcinoma, considering follicular thyroid carcinoma, skin biopsy was performed at two sites: a 4 mm punch biopsy from the subcutaneous nodule and an incisional biopsy from the scalp lesion (Fig. 4A).

The 4 mm punch biopsy showed bland-looking maturing epidermis and dermis. There were focal deposits of hyperchromatic round-to-oval cells with eosinophilic cytoplasm arranged in cohesive aggregates within the subcutaneous tissue. The focal area of vascular invasion was seen, and the incisional biopsy showed bland-looking epidermis. Dermal subcutaneous deposits of cohesive aggregates of pleomorphic round cells with eosinophilic cytoplasm are shown in Fig. 4A, B.

Immunohistochemistry was recommended, but the test was not available. The patient had no willingness for further treatment owing to cultural constraints. She preferred going to holy water places seeking spiritual intervention rather than medical treatment. Finally, the patient was diagnosed with cutaneous metastasis from presumed poorly differentiated thyroid carcinoma, considering the top differential diagnosis, thyroid carcinoma. She was referred to the Oncology Unit of Black Lion Hospital. The timeline was as follows: T0 = symptom duration 6 months, T1 = diagnosis was made within 3 weeks (results of the biopsy were awaited), T2 = death occurred 3 months after presentation. For this patient, total thyroidectomy and sending the sample for biopsy were indicated, but owing to the challenge of the long wait list for patients to undergo surgery, we could not perform thyroid biopsy. The patient passed away before receiving any treatment.

## Discussion and conclusion

Cutaneous metastases from thyroid cancer are uncommon. They typically arise in the setting of diffuse neoplastic illness. Some studies have reviewed the English literature from 1964 onward and discovered 43 cases of thyroid cancer with skin metastases [8, 9]. Metastasis to the skin occurs because of lymphatic or hematogenous dissemination of the tumor. The most common sources of cutaneous metastases are, in generally accepted order of frequency: breast, melanoma, lung, colon, stomach, upper aerodigestive tract, uterus, and kidney [10]. Thyroid cancer is the most common type of endocrine-related cancer. Spread of thyroid cancer outside the neck (metastases) is rare, occurring in between 1.2% and 13% of patients [11–13]. Metastatic sites include cervical lymph nodes, lungs, and bone [14]. Metastases to the brain, breast, liver, kidney, muscle, and skin are rare [15].

The classic clinical presentation of cutaneous metastasis is a rapid onset of solitary or multiple, asymptomatic, skin-colored, mobile, firm, round or oval nodule(s). The color varies, from skin-colored to pink–red to blue–black. Additional presentations could be erythematous patches that resemble erysipelas or dermatitis (carcinoma erysipeloides), indurated plaques, dermal infiltration causing sclerosis (carcinoma en cuirasses), vascular changes (carcinoma telangiectodes), dermatitis-like appearance of Paget’s disease, pyogenic granuloma-like, reticulated vascular patterns, and Zosteriform.

Diagnosis is based on clinical and pathologic assessment of the skin involved. Histopathologic features help identify the source of the primary tumor. Microscopically, the collection of neoplastic cells, which usually resemble their malignancy of origin, is seen in the dermis and/or subcutaneous tissue. Additional features, such as tumor cells in an “Indian filing” pattern, lymph vascular invasion, necrosis, and a tumor-free “grenz zone,” are also helpful for the diagnosis of metastatic skin lesions. Immunohistochemical staining is often required to achieve the correct diagnosis in a presumed poorly differentiated carcinoma.

Treatment requires a multidisciplinary approach, including medical and surgical oncologists, radiation oncologists, and mental health care providers. Treatment follows the regimen appropriate for the primary metastatic malignancy. Removal of skin lesions by simple excision may enhance the patient’s quality of life but has little effect on the outcome that is dictated by the primary cancer. For functional, palliative, and cosmetic outcomes, local treatment is indicated. Local treatment options include imiquimod cream, liquid nitrogen cryotherapy, photodynamic therapy, excision, carbon dioxide laser therapy, pulsed dye laser therapy, topical and intralesional chemotherapy, and cytokines. When malodorous, topical metronidazole solution can be applied via cotton gauze or pump spray once to twice daily. Debridement can be performed if lesions bleed or crust.

Systemic therapies are indicated for immunotherapy- or chemotherapy-responsive tumors. Treatment follows the regimen appropriate for the primary metastatic malignancy. Electrochemotherapy combines the administration of nonpermeable or poorly permeable highly intrinsic cytotoxic drugs with the application of short and intense electric pulses to the tumors to facilitate drug delivery into the cancer cells.

The patient deferred follow-up and passed away after 3 months of initial presentation. Cutaneous metastases are usually suggestive of disseminated illness and have a poor prognosis. Thus, early diagnosis and therapy are necessary. Similar limitations are the diagnosis of secondary infiltrates of presumed poorly differentiated carcinoma. Owing to a lack of immunohistochemistry and inability to perform a thyroid biopsy, we could not explicitly state that this was cutaneous metastasis from thyroid carcinoma, but the physical examination and other diagnostics supported the high likelihood of this diagnosis.

## Data Availability

This published article contains all the data generated or analyzed during the present study.
